# Long-term changes in psychosocial well-being in children with obesity deciding not to participate in a lifestyle intervention

**DOI:** 10.1007/s40519-026-01840-w

**Published:** 2026-03-16

**Authors:** Camilla R. Benjaminsen, Jens M. Bruun, Malthe J. Pedersen, Loa Clausen, Jane N. Østergaard, Rasmus M. Jørgensen

**Affiliations:** 1 Steno Diabetes Center Aarhus, Aarhus University Hospital, Palle Juul-Jensens Blvd. 11, 8200 Aarhus N, Denmark; 2 Department of Clinical Medicine, Faculty of Health, Aarhus University, 8200 Aarhus N, Denmark; 3 Danish National Center for Obesity, 8200 Aarhus N, Denmark; 4 Department of Public Health, Aarhus University, 8000 Aarhus C, Denmark; 5 Department of Child and Adolescent Psychiatry, Aarhus University Hospital, Psychiatry, 8200 Aarhus N, Denmark; 6 Department of Pediatrics, Aarhus University Hospital, 8200 Aarhus N, Denmark

**Keywords:** Childhood obesity, Psychosocial well-being, Deciding not to participate, Lifestyle interventions, Pediatric health

## Abstract

**Purpose:**

To investigate whether children with obesity who decide not to participate in a lifestyle intervention experience a more negative long-term development in psychosocial well-being.

**Methods:**

This observational study included 403 children aged 5–10 years with obesity. Children who decided not to participate (*n* = 71) were compared to those not invited (not-invited group, *n* = 225) and those attending the lifestyle intervention (intervention group, *n* = 107). Psychosocial well-being was assessed with a mean follow-up of 24.5 ± 4.5 months using the self-reported Danish National Well-being Questionnaire. Data from national registries and mandatory health examinations were included. Within-group changes were analyzed using McNemar’s test, and group differences with multivariable logistic regression.

**Results:**

Adjusted analyses showed no significant differences in well-being changes between groups. However, children who decided-not-to-participate reported an overall decrease in psychosocial well-being with significantly increased school-related loneliness and reduced classroom concentration and helpfulness over time. Similarly, the not-invited group reported reduced school-related classroom concentration, helpfulness, and enjoyment but improved loneliness and bullying over time.

**Conclusions:**

Deciding not to participate in a lifestyle intervention for childhood obesity was associated with a more negative development in psychosocial well-being over time; however, causality needs to be determined.

*Clinical trial registration*: ClinicalTrials.gov identifier: NCT06705231.

Level of evidence: Level III, longitudinal cohort study.

**Supplementary Information:**

The online version contains supplementary material available at 10.1007/s40519-026-01840-w.

## Introduction

Childhood obesity is increasing worldwide and is associated with an increased risk of developing lifestyle diseases later in life, such as type 2 diabetes and cardiovascular disease [1–3]. While these complications typically appear later in adulthood, reduced psychosocial well-being often occurs during childhood [4]. Poor self-esteem is commonly reported among children living with obesity and is associated with higher levels of sadness and loneliness [5]. Furthermore, children living with obesity are at higher risk of experiencing bullying [6], have a lower quality of life (QoL) [7], and face a greater risk of depression compared to their peers [8]. Family-centered lifestyle interventions using a multifactorial approach are still considered the first-line treatment when addressing childhood obesity [9, 10]. The literature suggests that such interventions can stabilize or, to some extent, reduce body weight when treating children with obesity [10–13]. Furthermore, obesity interventions may have positive effects on psychosocial well-being in children and adolescents [14–19]. However, most of these studies do not include a reference group, and the longest follow-up is 12 and 18 months [17, 18]. Despite these potential benefits, several barriers may prevent caregivers from enrolling their children in lifestyle interventions. Factors such as life stressors, societal norms, and stigma related to body size, and negative prior experiences with the healthcare system are among the primary reasons found in previous studies [20–22]. However, no studies have yet explored whether deciding not to participate in a lifestyle intervention is associated with decreased psychosocial well-being of children with obesity. Gaining these insights is important to increase awareness of the needs of these children, and hopefully lead to more inclusive and tailored support strategies.

Since we previously reported that deciding not to participate in a lifestyle intervention for childhood obesity was without meaningful effects on long-term body mass index (BMI) z-score [23], the aim of this study was to investigate the long-term change in psychosocial well-being in these children. Using data from public primary schools, we examined within-group changes in psychosocial well-being from inclusion to follow-up, and compared children who decided-not-to-participate in a family-centered lifestyle intervention to those who attended the intervention, and those who were not invited to participate. A secondary aim was to investigate whether sex, BMI z-score at inclusion, parental education level, parental psychiatric illness, immigration status, or change in BMI z-score modified the effect of deciding not to participate when compared to the not-invited and intervention groups.

## Methods

### Study design

This observational cohort study included children with obesity living in Aarhus Municipality, Denmark, between August 1st, 2014, and June 30th, 2020. In Denmark, all children in public primary schools undergo mandatory clinical health examinations, including assessments of weight and height. In addition, these children annually complete a questionnaire regarding psychosocial well-being. This study combined data from these mandatory school health examinations, the Danish national registries, and the Danish National Well-being Questionnaire (DNWQ). Follow-up continued until February 1st, 2023.

### Participants

This study included children aged 5–10 years with obesity who either decided not to participate in a lifestyle intervention offered by Aarhus Municipality (decided-not-to-participate group), were not invited to participate (not-invited group), or attended the intervention (intervention group).

All children in Denmark will during primary school undergo mandatory health examinations, which include regular assessments of weight and height, performed by municipal health nurses. In this study, the children were identified during these examinations during school hours [12]. If a child’s BMI, adjusted for age and sex, exceeded the International Obesity Task Force (IOTF) threshold for obesity (iso-BMI ≥ 30 kg/m^2^) [24], the parents were notified, and families were invited to participate in the lifestyle intervention. Parental decisions regarding participation were recorded.

The inclusion criteria for this study were: (1) obesity, defined by the IOTF guidelines (iso-BMI ≥ 30 kg/m^2^, adjusted for age and sex) [24] at an inclusion visit between August 1st, 2014, and June 30th, 2020; (2) age 5–10 years at the inclusion visit; (3) a completed DNWQ at inclusion within a timeframe of 6 months before to 6 months after inclusion (for the deciding-not-to-participate- and not-invited groups) or within a timeframe of 10 months before to 2 months after inclusion (for the intervention group), ensuring a sufficiently large sample while minimizing intervention effects on baseline data; and (4) a completed DNWQ at follow-up (1–3 years after the inclusion visit).

**Fig. 1:**
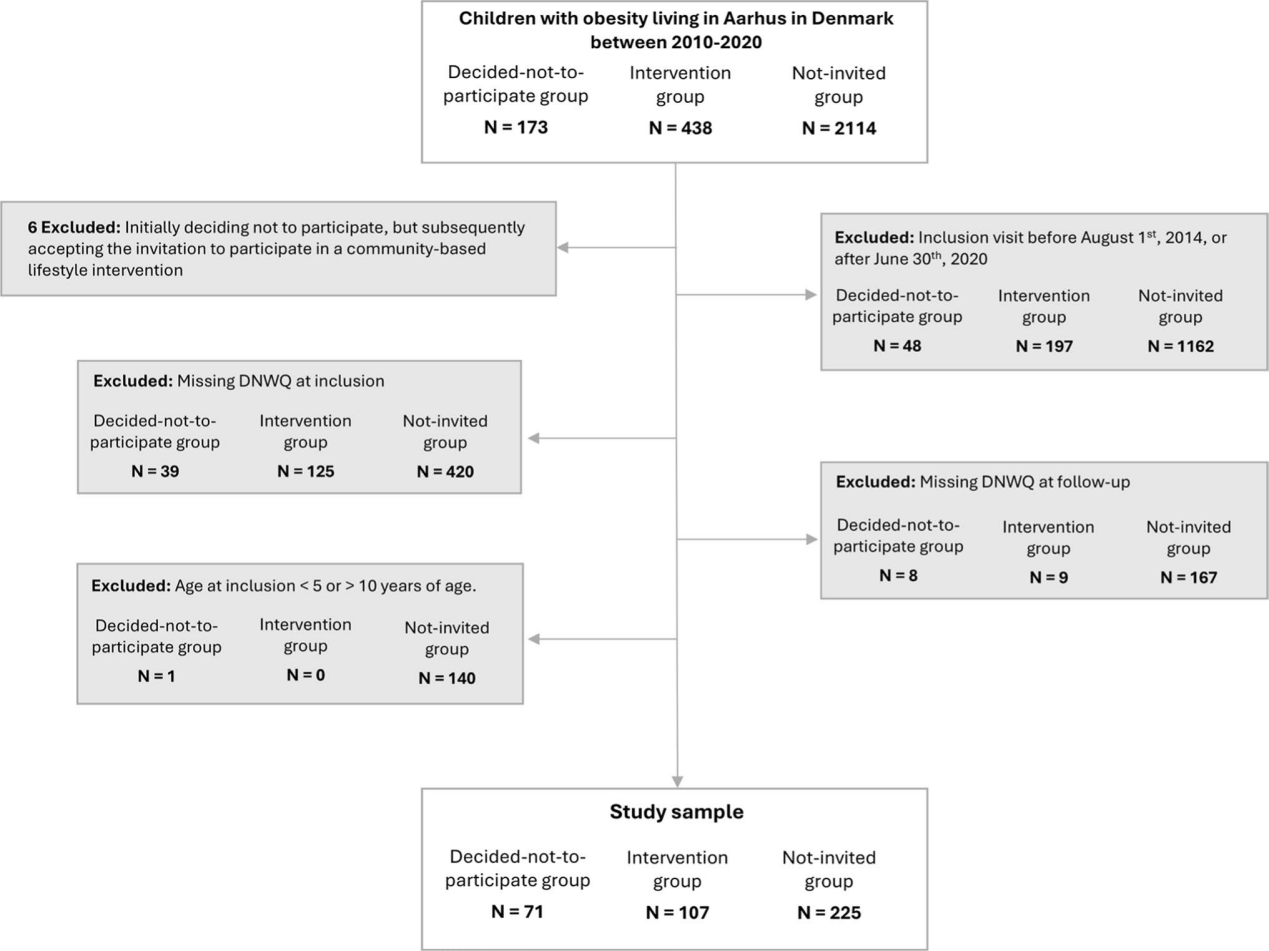
Flow chart of the study sample

The exclusion criterion was children who initially decided-not-to-participate but later accepted the intervention (Fig. 1).

The inclusion visit was defined as the observation containing height and weight closest to the day of deciding not to participate (decided-not-to-participate group), the first day of attending the intervention (intervention group), or the first observation of obesity (not-invited group).

### Exposure/the groups

#### The deciding not-to-participate group:

The decided-not-to-participate group consisted of children with obesity residing in Aarhus Municipality who were invited to participate in the lifestyle intervention mentioned below but, for unknown reasons, decided-not-to-participate.

#### The intervention group:

The intervention group consisted of children with obesity who participated in a lifestyle intervention in Aarhus Municipality. As previously described, the intervention was a 1-year family-centered, multicomponent behavioral lifestyle program designed for children aged 5–8 years living with obesity [12]. The program aimed to educate parents on adopting and sustaining a healthier lifestyle, with parents playing a central role in implementing intervention strategies between consultations. The intervention was tailored to the individual needs of each child and focused on promoting lifestyle changes related to nutrition, eating habits, physical activity, screen time, sleep, and mental health. The intervention was delivered by municipal health nurses with expertise in dealing with childhood obesity, either at local healthcare centers or through home visits, and consisted of three to four consultations scheduled with predefined intervals. During these consultations, the nurses employed methods such as mind mapping, dialoge wheels, and motivational interviewing to explore and support changes in the family’s daily routines related to lifestyle behaviors, with a particular emphasis on parental responsibility, as well as the well-being and mental health of both the child and the family. In addition, participants were invited to attend complimentary, supervised physical activity sessions on a weekly basis.

#### The not-invited group:

The not-invited group comprised all identified children with obesity living in Aarhus Municipality who were not invited to participate in the intervention. Why these children were not invited to participate in the intervention is unknown. The demographic characteristics of this group, as well as the differences compared to the decided-not-to-participate group and the intervention group, are presented in Table 1.

**Table 1:** Participant characteristics at inclusion, stratified by group

	Decided-not-to-participate group (*n* = 71)	Intervention group (*n* = 107)	Not-invited group (*n* = 225)
Age (years) at inclusion, median (q1, q3)	6.7 (6.5, 6.9)	7.0 (6.7, 7.6)	7.2 (6.6, 8.1)
BMI z-score, median (q1, q3)	3.0 (2.7, 3.4)	2.9 (2.5, 3.2)	2.9 (2.6, 3.1)
Follow-up time (months), mean ± SD	25.1 ± 4.8	24.1 ± 4.9	24.4 ± 4.1
Sex, % (*n*)			
Boys	53.5% (38)	45.8% (49)	48.9% (110)
Girls	46.5% (33)	54.2% (58)	51.1% (115)
Highest completed household education, % (*n*)			
Short	23.1% (15)	17.0% (18)	17.0% (38)
Middle	50.8% (33)	47.2% (50)	54.0% (121)
Long	26.2% (17)	35.8% (38)	29.0% (65)
Family type, % (*n*)			
Non-two-parent families	32.4% (23)	40.6% (39)	34.4% (66)
Two-parent families	67.6% (48)	59.4% (57)	65.6% (126)
Immigration status, % (*n*)			
Danish origin	63.4% (45)	55.2% (53)	65.6% (126)
Non-Danish	36.6% (26)	44.8% (43)	34.4% (66)
Parental psychiatric illness, % (*n*)			
No	80.3% (57)	71.0% (76)	61.3% (138)
Yes	19.7% (14)	29.0% (31)	38.7% (87)
Psychiatric diagnosis, child, % (*n*)			
No	93.0% (66)	95.3% (102)	94.7% (213)
Yes	7.0% (5)	4.7% (5)	5.3% (12)
Q1—Enjoyment at school, % (*n*)			
Poor well-being	18.3% (13)	25.2% (26)	20.7% (46)
Good well-being	81.7% (58)	74.8% (77)	79.3% (176)
Q2—Feelings of loneliness at school, % (*n*)			
Poor well-being	32.9% (23)	47.2% (50)	53.5% (116)
Good well-being	67.1% (47)	52.8% (56)	46.5% (101
Q3—Experiences of bullying, % (*n*)			
Poor well-being	41.4% (29)	61.5% (64)	59.5% (128)
Good well-being	58.6% (41)	38.5% (40)	40.5% (87)
Q4—Experiences of stomach aches, % (*n*)			
Poor well-being	42.0% (29)	49.5% (53)	41.8% (92)
Good well-being	58.0% (40)	50.5% (54)	58.2% (128)
Q5—Problem-solving, % (*n*)			
Poor well-being	48.6% (34)	54.8% (57)	60.8% (132)
Good well-being	51.4% (36)	45.2% (47)	39.2% (85)
Q6—Concentration in the classroom, % (*n*)			
Poor well-being	33.3% (23)	43.4% (46)	43.1% (94)
Good well-being	66.7% (46)	56.6% (60)	56.9% (124)
Q7—Helpfulness in the classroom, % (*n*)			
Poor well-being	20.6% (14)	21.4% (22)	35.0% (76)
Good well-being	79.4% (54)	78.6% (81)	65.0% (141)

*q1,q3* Interquartile range, *BMI* Body mass index

### Outcome

The outcome of this study was the responses to seven selected items from the DNWQ. Introduced in 2015, the DNWQ evaluates how primary school children perceive their well-being and learning environment at school. It is administrated annually between January 20th and March 20th as a part of the curriculum for all children in public primary schools, achieving a high response rate of 85% [25]. The DNWQ is completed independently using the unique identification number assigned to all Danish residents [26]. For pupils in kindergarten class to grade 3, the DNWQ comprises 20 items rated on a three-point Likert scale (1–3) to measure their level of agreement with each item, while pupils in grades 4–9 complete 40 items rated on a five-point Likert scale (1–5) [27]. For both scales, 1 represents the lowest possible psychosocial well-being, while 3 (kindergarten class to grade 3) and 5 (grades 4–9) indicate the highest possible well-being (see Table S1).

Seven items (Q1–Q7) were selected to assess psychosocial well-being in this study, chosen collaboratively by the research team for their relevance in capturing key dimensions of children’s well-being and ensuring that they accurately represented the children’s overall psychosocial well-being while maintaining comparability across the two age-specific versions of the DNWQ (Table S1). The items focused on enjoyment at school, feelings of loneliness at school, experiences of bullying, stomach aches, problem-solving skills, concentration in the classroom, and helpfulness towards classmates. Responses were dichotomized into good and poor well-being. Responses 1 and 2 (kindergarten class to grade 3) and 1–3 (grades 4–9) were considered related to poor well-being.

The dichotomized responses from the time of inclusion and follow-up were used to assess changes within groups over time, as well as differences between groups, prioritizing the follow-up responses recorded closest to 2 years after inclusion.

### Data sources and covariates

Data for this study were obtained from TM-Sund, a data-capturing tool used by health nurses in Aarhus Municipality to store height and weight measurements recorded during the mandatory school health examinations. Data on height and body weight were used to identify children with obesity, as defined by the IOTF cutoff criteria [24]. Furthermore, TM-Sund provided information on whether caregivers accepted or decided not to participate in the intervention. For each child included, additional height and weight measurements from mandatory health examinations were extracted. Data from the DNWQ were obtained from the Danish Agency for IT and Learning (STIL), Ministry of Education [27]. The DNWQ were linked to the cohort at Statistics Denmark using the unique identification numbers [26].

Data on potential confounders, including parental educational level, immigration status, family structure, and psychiatric diagnoses at the time of inclusion, were extracted from registers at Statistics Denmark (DST) [28–30].

Parental educational level was determined by the highest completed education within the household and categorized into: “short” (primary school, UNESCO’s International Standard Classification of Education (ISCED) levels 1–2), “middle” (high school, vocational education, and similar shorter educations, ISCED levels 3–5), and “long” (bachelor’s degree or higher, ISCED levels 6–8) [31]. Immigration status was classified as “Danish” for children of ethnic Danish origin and “non-Danish” for children born outside Denmark or with foreign-born parents [32]. Family structure was categorized as living in “two-parent families” and “non-two-parent families” [33]. Data regarding psychiatric diagnoses of the children and their parents were obtained from the Danish National Patient Register. Parental psychiatric illness was defined as having at least one parent with a registered psychiatric diagnosis at inclusion [34] and was included as an indicator of familial psychiatric risk. The child was classified as having a psychiatric diagnosis at inclusion if any prior psychiatric diagnosis had been recorded [35].

### Statistical analyses

McNemar’s test was applied to evaluate within-group changes in psychosocial well-being (Q1–Q7) from inclusion to follow-up. To compare the change in psychosocial well-being between groups, we used two logistic regression models: a univariable model adjusting only for well-being at inclusion, and a multivariable model that adjusted for well-being at inclusion, BMI z-score at inclusion, sex, parental educational level, and immigration status. The models were limited to one covariate per 10 observations of the least common outcome. Participants who completed the DNWQ but omitted the specific item of interest were excluded from the respective analysis.

To explore the effect independent of weight change, a second multivariable logistic regression model was conducted, adjusted for change in BMI z-score, including only participants with a BMI z-score measured within 6 months of the DNWQ at follow-up. The model included well-being at inclusion, BMI z-score change, BMI z-score at inclusion, and sex as covariates.

Multiple imputation with chained equations was applied to replace missing values for parental education level (*n* = 8 [2.0%]), immigration status (*n* = 44 [10.9%]), and family type (*n* = 44 [10.9%]). Rubin’s rule was applied to pool results of 100 imputed datasets [36].

To conduct analyses of effect modification for items Q1–Q4, we used the multivariable logistic regression analyses adjusted for possible confounders and included the fixed effect (decided-not-to-participate group) applied to the dichotomized variables of sex (girls vs. boys), BMI z-score (< 50th percentile vs. > 50th percentile of the BMI z-score distribution), parental educational level (ISCED levels 1–4 vs. ISCED levels 5–8), immigration status (Danish vs. non-Danish), family psychiatric diagnosis (child or parental psychiatric diagnosis: yes vs. no), and BMI z-score change (reduction vs. increase).

All analyses were conducted using STATA 15 (College Station, TX, USA: StataCorp LLC). Results are presented with 95% confidence intervals (CIs), and a significance level of 5% was applied.

## Results

### Inclusion

A total of 403 children were included in this study: 71 children in the decided-not-to-participate group, 107 children in the intervention group, and 225 children in the not-invited group (Fig. 1). The mean (SD) follow-up time was 24.5 ± 4.5 months, with no significant differences between the groups (Table 1). At inclusion, a higher proportion of children in the not-invited group had at least one parent with psychiatric illness compared to the decided-not-to-participate group (*p* < 0.01) (Table 1). Although non-significant, the decided-not-to-participate group tended to have slightly lower parental education levels and fewer immigrant families compared to the intervention group.

Children in the decided-not-to-participate group were significantly less likely to experience loneliness and bullying at inclusion compared to the not-invited group (both *p* < 0.01). In addition, bullying was 20% more prevalent at inclusion among children who accepted the intervention than among those who decided-not-to-participate (*p* = 0.01). A significantly higher proportion of children in the decided-not-to-participate group reported experiencing helpfulness in the classroom compared to the not-invited group (*p* = 0.03) (Table 1).

BMI z-scores at follow-up were available for 39 children in the decided-not-to-participate group, 58 children in the intervention group, and 77 children in the not-invited group. During follow-up, we observed mean reductions of 0.10 SD in BMI z-scores for the decided-not-to-participate and the not-invited groups, and a reduction of 0.21 SD for the intervention group with no difference between the groups (*p* = 0.41).

### Changes in psychosocial well-being during follow-up

**Fig. 2:**
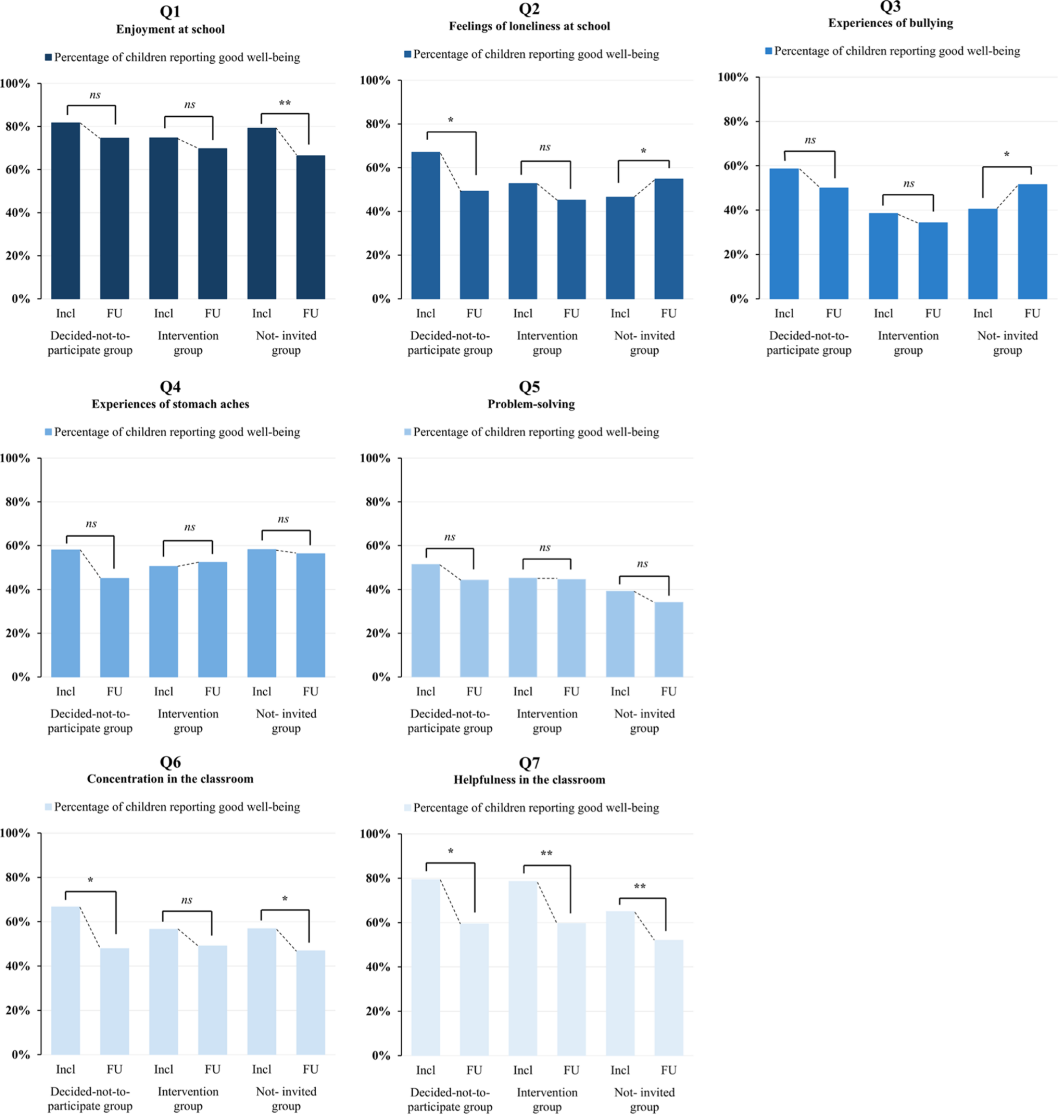
Percentage of children reporting good well-being in the items on psychosocial well-being at inclusion and at follow-up, by the decided-not-to-participate group, the intervention group, and the not-invited group. McNemar’s test was employed to assess changes in psychosocial well-being from inclusion to follow-up for each item (Q1–Q7) in all three groups. ns indicates non-significant (*p* > 0.05), * indicates *p* ≤ 0.05, and ** indicates *p* ≤ 0.01 for each comparison (inclusion vs. follow-up)

As illustrated in Fig. 2, children in the decided-not-to-participate group experienced impairments in psychosocial well-being across all seven items, with a significant increase in loneliness at school (Q2) and reduction in concentration (Q6) and helpfulness in the classroom (Q7) during follow-up. In the intervention group, a significant reduction in well-being was observed only for helpfulness in the classroom (Q7). Children in the not-invited group demonstrated bi-phasic changes with a significant decrease in enjoyment at school (Q1), classroom concentration (Q6), and helpfulness in the classroom (Q7), alongside significant improvements in loneliness at school (Q2) and experiences of bullying (Q3).

### The deciding-not-to-participate group compared to the not-invited group

**Fig. 3:**
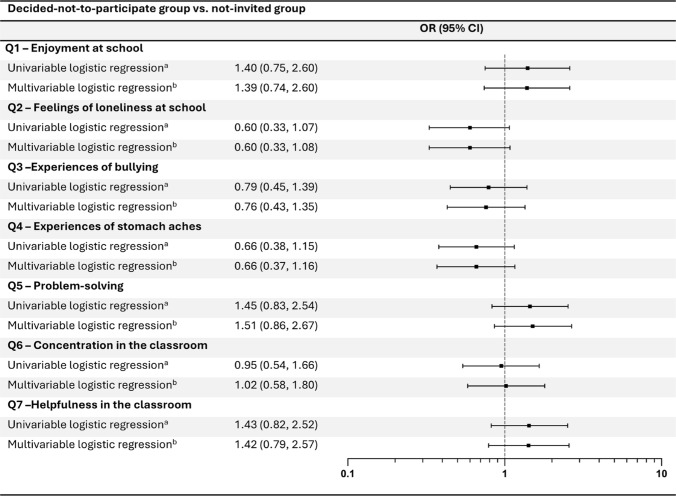
Comparison between the decided-not-to-participate group and the not-invited group. Logistic regression models comparing well-being at follow-up between the decided-not-to-participate group and the not-invited group for all items (Q1–Q7). Odds ratios (ORs) greater than 1.0 indicated more favorable outcomes for the children in the decided-not-to-participate group compared with the not-invited group. ^a^Logistic regression model adjusted for well-being at inclusion. ^b^Logistic regression model adjusted for well-being at inclusion, BMI z-score, sex, parental educational level, and immigration status

Comparing the change in psychosocial well-being during follow-up between the decided-not-to-participate group and the not-invited group, neither the univariable nor the multivariable logistic regression models revealed significant differences for any of the included items (Q1–Q7) (Fig. 3). Similar findings were observed after adjusting for change in BMI z-score (Fig. S1A).

BMI z-score at inclusion was identified as an effect modifier for bullying (Q3), with significant differences in effects (*p* = 0.05) observed between children in the lower 50% of the BMI z-score distribution [1.44 (0.57, 3.65)] and those in the upper 50% [0.52 (0.24, 1.15)] (Fig. S2). In addition, sex also modified the association between deciding not to participate and bullying, with a significantly stronger association observed among boys [0.42 (0.19, 0.92)] than girls [1.53 (0.63, 3.75)] (Fig. S2).

### The deciding-not-to-participate group compared to the intervention group

**Fig. 4:**
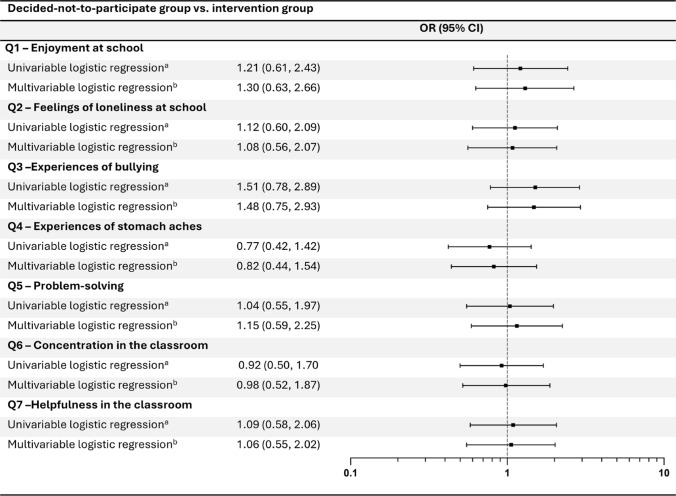
Comparison between the decided-not-to-participate group and the intervention group. Logistic regression models comparing well-being at follow-up between the decided-not-to-participate group and the intervention group for all items (Q1–Q7). Odds ratios (ORs) greater than 1.0 indicated more favorable outcomes for the children in the decided-not-to-participate group compared with the intervention group. ^a^Logistic regression model adjusted for well-being at inclusion. ^b^Logistic regression model adjusted for well-being at inclusion, BMI z-score, sex, parental educational level, and immigration status

No differences in change in psychosocial well-being during follow-up were observed when comparing the decided-not-to-participate group with the intervention group for any of the items (Q1–Q7) in neither the univariable nor the multivariable logistic regression model (Fig. 4). Adjusting for change in BMI z-score revealed similar results (Fig. S1B).

Sex and immigration status were identified as effect modifiers for the outcomes of loneliness and bullying; however, the wide CIs indicate considerable uncertainty (Fig. S3).

## Discussion

This study provides novel insights into the long-term changes in psychosocial well-being of children with obesity who decided-not-to-participate in a community-based lifestyle intervention. While logistic regression models did not reveal significant differences between groups, children who decided-not-to-participate experienced a decline in overall psychosocial well-being over a 2-year period. This decline was particularly evident in increased loneliness at school and reduced classroom concentration and helpfulness, despite an unchanged degree of obesity. In contrast, children in the not-invited group also showed declines in classroom concentration, helpfulness, and enjoyment at school but reported reduced loneliness and bullying, showing a distinct pattern compared to the decided-not-to-participate group. In addition, children in the intervention group reported a significant decline in classroom helpfulness.

These findings suggest that children who decide not to participate in an intervention for childhood obesity may experience a more negative development in psychosocial well-being over time.

To our knowledge, this is the first study to explore psychosocial well-being in children who decide not to participate in a lifestyle intervention focusing on treatment of children living with obesity. While previous research primarily has focused on body weight (i.e., BMI z-score) [23], little is known about the long-term changes in well-being of these children, with caregivers rejecting participation. Understanding this group is crucial, as this decision may have implications for their long-term psychosocial and mental well-being.

Although we found no studies directly comparable to the present, the observed impairments in psychosocial well-being align with existing evidence linking obesity to loneliness [5], impaired QoL [7], and reduced social and school functioning [37]. In addition, the observed improvements in psychosocial well-being (i.e., loneliness and bullying) in the not-invited group, which were not seen in the decided-not-to-participate group, suggest that declining participation may be associated with long-term implications for well-being. A finding that cannot be explained by changes in BMI, as the groups experienced similar reductions in BMI z-score [23]. The reasons behind these differences remain uncertain and may be multifaceted. It is possible that children in the not-invited group came from families with more resources (e.g., psychosocial), leading health professionals to consider them less in need of intervention. Conversely, studies suggest that some children are not offered interventions due to concerns about parental challenges, including mental health difficulties [38]. In line with this, the not-invited group in this study had the highest proportion of children with parental psychiatric illness. In addition, logistical barriers such as distance to intervention sites or alternative referral pathways may have influenced participation. While our baseline characteristics did not indicate that the decided-not-to-participate group was more challenged than the other groups, it is possible that unmeasured factors contributed to their decision and subsequent development in well-being [20]. Moreover, the observed deterioration in psychosocial well-being in the decided-not-to-participate group may also be interpreted as regression towards the mean, as this group exhibited the most favorable psychosocial well-being at baseline. Consequently, some of the observed decline at follow-up may, therefore, reflect natural variation rather than a true adverse effect of declining participation.

Positive effects on psychosocial well-being have previously been reported for children engaging in lifestyle interventions [14, 15, 18, 39]. Despite these positive effects, it is important to recognize that some caregivers will choose not to enroll their children in such interventions. Limited knowledge exists on why families decide not to participate; however, factors such as life stressors, stigma regarding body size, societal norms, and negative experiences with the healthcare system are the main determinants and reasons reported [20–22]. Interestingly, this study found that children in the decided-not-to-participate group had a lower prevalence of parental psychiatric illness and overall better psychosocial well-being at inclusion than those in the not-invited group. An explanation may be that caregivers without psychiatric challenges experience fewer daily life stressors, making them feel more confident in managing their child’s weight independently. However, the parents may not take the necessary steps to address it effectively, potentially contributing to the observed declines in long-term psychosocial well-being. Furthermore, although no significant differences were observed, the decided-not-to-participate group tended to have slightly lower parental education levels and fewer immigrant families compared to the intervention group. This aligns with previous research reporting that mothers with a university education were nearly three times more likely to enroll their child in an intervention than those without tertiary education [22].

Notably, children in the decided-not-to-participate group exhibited an overall better psychosocial well-being at inclusion compared to those in the intervention and not-invited groups. This could suggest that families who perceived their child’s well-being as relatively good were less motivated to engage in the intervention. In addition, bullying was 20% more prevalent at inclusion among children who accepted the intervention than among those who decided-not-to-participate, indicating that children experiencing greater social challenges were more likely to engage in the intervention.

In contrast to previous reports of improvements in psychosocial well-being following participation in lifestyle interventions [14, 15, 39, 40], we did not observe significant improvements in psychosocial outcomes in the intervention group over the 2-year follow-up. Apart from a decline in perceived helpfulness in the classroom, psychosocial well-being remained largely unchanged among children who participated in the intervention. Several factors may explain this finding. First, the long follow-up period may have attenuated potential initial benefits of the intervention, as effects on psychosocial well-being may be time-limited only. In support, our previous study of the same intervention demonstrated a rebound in BMI z-score during the 6–12-month follow-up, following the initial post-intervention improvements [12], suggesting that early benefits may not be sustained and could adversely affect the motivation for maintaining lifestyle changes. Second, it is possible that the intervention was not sufficiently intensive or specifically targeted to induce measurable and lasting changes in psychosocial well-being, as its primary focus was on lifestyle and weight-related behaviors rather than psychosocial functioning.

In this study, BMI z-score at inclusion and sex modified the association between the exposure and the degree of bullying when comparing the decided-not-to-participate group with the not-invited group. This may suggest that deciding not to participate in a lifestyle intervention may be more detrimental for children with a BMI z-score in the upper 50% of the distribution and for boys, potentially exacerbating their vulnerability to bullying and psychosocial distress. Boys have earlier been shown to experience greater improvements in QoL following participation in lifestyle interventions but are also less likely to attend weight management programs compared to girls, underscoring the need for tailored approaches to enhance their engagement [15, 41].

All Danish residents have access to free universal healthcare, representing some degree of social equality and may explain the lack of significant differences between the groups [42]. Equitable access to essential services and a relatively homogenous society may reduce socioeconomic disparities and their impact on health outcomes, potentially mitigating the negative consequences of declining participation [42]. However, further research is needed.

Our findings suggest that children whose families choose not to participate in lifestyle interventions may be particularly vulnerable to unfavorable psychosocial development. Future research should focus on developing and evaluating interventions that integrate psychological support within obesity treatment programs by reducing barriers to engagement and focusing on subjects with impaired psychosocial well-being. In particular, low-threshold, family-based psychological approaches addressing social functioning, emotional well-being, and school-related challenges may be especially relevant. In addition, systematic identification of psychosocial difficulties and family-related risk factors may facilitate more tailored preventive strategies for children who are not offered or do not engage in treatment.

### Strengths and limits

A key strength of this study is the unique combination of data sources, including annual psychosocial well-being measures from schools, national registry data, and municipal records, enabling an extended follow-up period and the inclusion of a reference group.

Denmark’s comprehensive national registries, linked through unique social security numbers, enable adjustment for potential confounders, while the obligatory school-based questionnaire ensures a high response rate (85–90%), minimizing selection and information bias [43]. In addition, Aarhus Municipality’s retention of records for children who decided not to participate in their intervention provides a rare opportunity to examine this specific group. These factors collectively make this study highly distinctive and offer valuable insights that would be difficult to obtain in other settings.

This study is limited by its reliance on self-reported measures of psychosocial well-being. While self-reports are generally accepted as the most reliable method for assessing subjective outcomes like well-being, the reliability of responses may vary with age-related comprehension levels [44]. However, evidence suggests that children as young as six can provide valid responses to health-related questionnaires [44]. Although most of the items used to assess psychosocial well-being in this study are well-validated, others lack validation, which could introduce uncertainty into the findings [27, 45]. Furthermore, the accuracy of the selected cutoff values is uncertain, which may introduce a risk of misclassification [27, 45]. In addition, the relatively small and homogenous sample size from one municipality in Denmark could limit the generalizability of the findings. Missing BMI z-scores at follow-up reduced the statistical precision for the secondary outcome and a skewed distribution may also introduce bias. The multivariable models could not account for all potential confounders due to the limited sample size, which might have affected the robustness of the results. Why the children in the not-invited group were not invited to participate remains unclear. Robson et al. reported that up to 20% of eligible children were not referred to interventions, often because the providers did not perceive the child as having obesity or doubted the family’s ability or willingness to engage in the program [38]. While this may influence which children receive an invitation, it is unlikely to have introduced substantial bias.

## Conclusion

Children who decided-not-to-participate in a lifestyle intervention exhibited an overall more pronounced decline in long-term psychosocial well-being over a 2-year period than what was observed among children who were not invited and children attending the intervention, despite similar long-term changes in BMI z-score.

## What is already known on this subject?

Family-centered lifestyle interventions are considered the first-line treatment when addressing childhood obesity. However, some caregivers choose not to enroll their children, even when the children meet all eligibility criteria. Factors such as life stressors, societal norms, and stigma related to body size, and negative prior experiences with the healthcare system are the primary reasons cited as to why caregivers decide not to let their children participate in lifestyle interventions. However, limited knowledge exists regarding the long-term consequences of this decision.

## What your study adds?

This study examines long-term changes in psychosocial well-being among children with obesity who chose not to participate in a family-centered lifestyle intervention, compared to those who attended the intervention, and those who were not invited. The findings suggest that children who decide not to participate in a childhood obesity intervention may experience a more negative development in psychosocial well-being over time. This highlights the importance of exploring alternative support options for families who choose not to participate.

## Supplementary Information

Below is the link to the electronic supplementary material.

Supplementary material 1.

## Data Availability

The data underlying this study are not publicly accessible due to restrictions imposed by the Danish General Data Protection Regulation. Researchers seeking access to the data may contact the corresponding author, CRB.
