# Supplementary material for: Long-term changes in psychosocial well-being in children with obesity deciding not to participate in a lifestyle intervention
Source: Eat Weight Disord. 2026 Mar 16;31(1):39. doi: 10.1007/s40519-026-01840-w (PMC13102802; doi:10.1007/s40519-026-01840-w)
Supplement: Supplementary file 1 — Supplementary material 1. [file 40519_2026_1840_MOESM1_ESM.pdf]

Journal: Eating and Weight Disorders - Studies on Anorexia, Bulimia and Obesity

Title

Long-term changes in psychosocial well-being in children with obesity deciding not to participate in a lifestyle intervention

Authors

Camilla R Benjaminsen[1] **(corresponding author)** (ORCID: 0009-0000-6104-1314)

Jens M Bruun[1,2,3] (ORCID: 0000-0001-9937-5467)

Malthe J Pedersen[4] (ORCID: 0000-0003-1510-3179)

Loa Clausen[2,5] (ORCID: 0000-0002-4559-8347)

Jane N Østergaard[1] (ORCID: 0000-0002-0627-1237)

Rasmus M Jørgensen[1,6] (ORCID: 0000-0001-9910-8924)

[1]Steno Diabetes Center Aarhus, Aarhus University Hospital, 8200 Aarhus N, Denmark
[2]Department of Clinical Medicine, Faculty of Health, Aarhus University, 8200 Aarhus N, Denmark
[3]Danish National Center for Obesity, 8200 Aarhus N, Denmark
[4]Department of Public Health, Aarhus University, 8000 Aarhus C, Denmark
[5]Department of Child and Adolescent Psychiatry, Aarhus University Hospital, Psychiatry, 8200 Aarhus N, Denmark
[6]Department of Pediatrics, Aarhus University Hospital, 8200 Aarhus N, Denmark

Contact information: Camilla Raaby Benjaminsen, Steno Diabetes Center Aarhus, Aarhus University Hospital, Palle Juul-Jensens Blvd. 11, 8200 Aarhus N, Denmark. Mail: 201709516@post.au.dk / cabenj@rm.dk

Table S1: The items from the Danish National Well-being Questionnaire (DNWQ).

Items on psychosocial well-being

Q1

Are you happy with your school? (kindergarten class to grade 3)

1	2	3
No	Yes, a little	Yes, very
☐	☐	☐

Are you happy with your school? (grades 4 to 9)

1	2	3	4	5
Never	Rarely	Occasionally	Often	Very often
☐	☐	☐	☐	☐

Q2

Do you feel lonely at school? (kindergarten class to grade 3)

1	2	3
Yes, often	Yes, sometimes	No
☐	☐	☐

Do you feel lonely? (grades 4 to 9)

1	2	3	4	5
Very often	Often	Occasionally	Rarely	Never
☐	☐	☐	☐	☐

Q3

Is anyone teasing you so that you feel sad? (kindergarten class to grade 3)

1	2	3
Yes, often	Yes, sometimes	No
☐	☐	☐

Have you been bullied this school year? (grades 4 to 9)

1	2	3	4	5
Very often	Often	Occasionally	Rarely	Never
☐	☐	☐	☐	☐

Q4

Does your stomach ache when you are at school? (kindergarten class to grade 3)

1	2	3
Yes, often	Yes, sometimes	No
☐	☐	☐

How often does your stomach ache? (grades 4 to 9)

1	2	3	4	5
Very often	Often	Occasionally	Rarely	Never
☐	☐	☐	☐	☐

Q5

Are you good at solving your problems? (kindergarten class to grade 3)

1	2	3
No	Yes, sometimes	Yes, mostly
☐	☐	☐

How often can you find a solution to problems, just by trying hard enough? (grades 4 to 9)

1	2	3	4	5
Never	Rarely	Occasionally	Often	Very often
☐	☐	☐	☐	☐

Q6

Can you concentrate during class? (kindergarten class to grade 3)

1	2	3
No	Yes, sometimes	Yes, mostly
☐	☐	☐

Can you concentrate during class? (grades 4 to 9)

1	2	3	4	5
Never	Rarely	Occasionally	Often	Very often
☐	☐	☐	☐	☐

Q7

Are you good at helping each other in class? (kindergarten class to grade 3)

1	2	3
No	Yes, a little	Yes, very
☐	☐	☐

Most students in my class are friendly and helpful. (grades 4 to 9)

1	2	3	4	5
Strongly disagree	Disagree	Neither agree nor disagree	Agree	Strongly agree
☐	☐	☐	☐	☐

Fig. S1: Comparison of psychosocial well-being at follow-up, independent of weight change, between the groups.

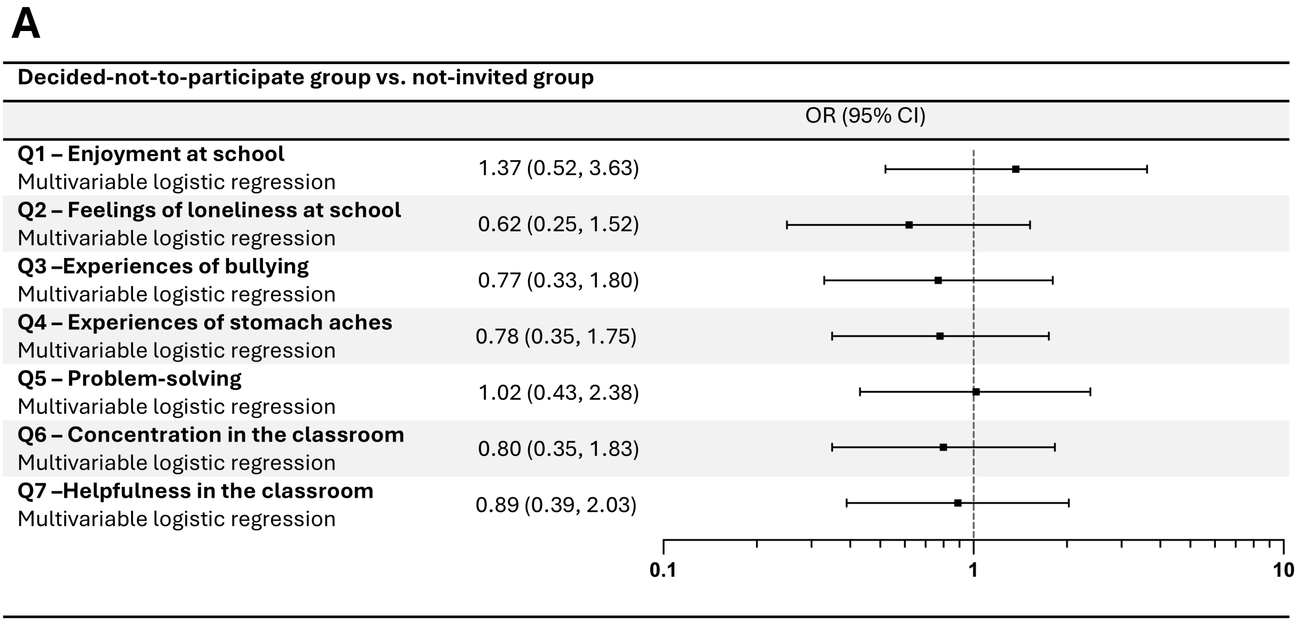

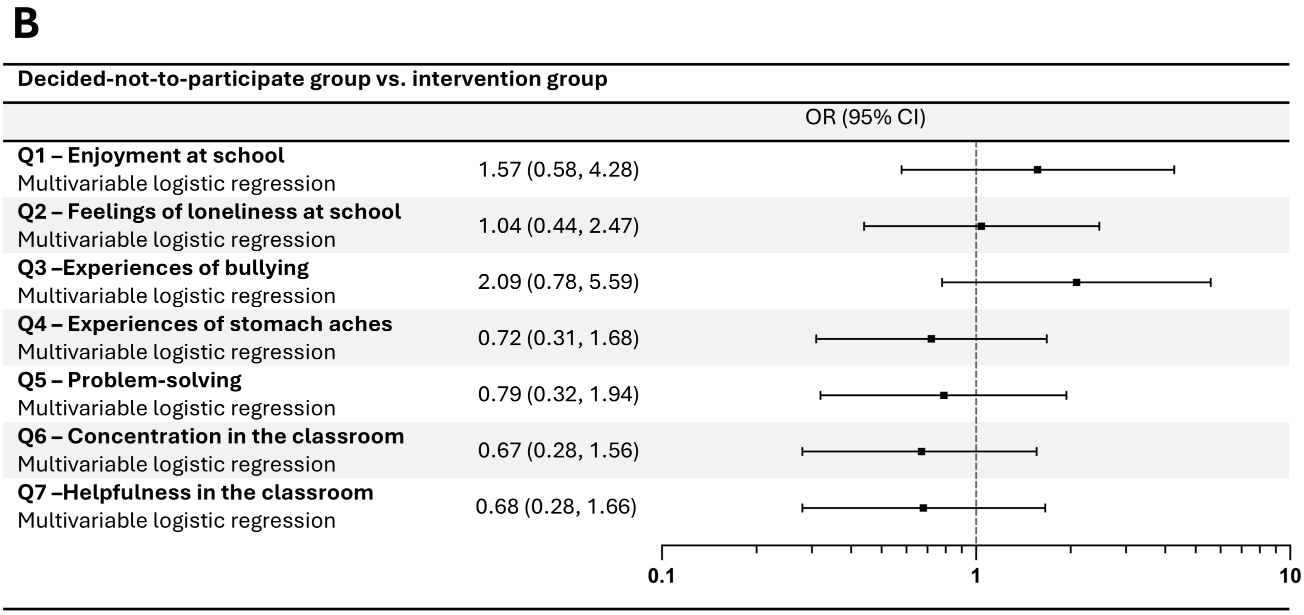

Logistic regression model comparing well-being at follow-up, independent of change in BMI z-score, between the decided-not-to-participate group and the not-invited group (A), and the decided-not-to-participate group and the intervention group (B).
Odds ratios (ORs) greater than 1.0 indicated more favorable outcomes for the children in the decided-not-to-participate group compared to those in the not-invited group (A) and the intervention group (B)
The model was adjusted for well-being at inclusion, BMI z-score change (from inclusion to follow-up), BMI-score at inclusion, and sex. OR, Odds ratio; CI, Confidence interval; BMI, Body mass index.

Fig. S2: Effect modification analyses comparing the psychosocial well-being between the decided-not-to-participate group and the not-invited group.

	Decided-not-to-participate group (N)	Not-invited group (N)	OR (95% CI)	P-value
**Q1 – Enjoyment at school**				
Sex[i]				0.72
Girls	33	113	1.23 (0.50, 3.00)	
Boys	38	107	1.56 (0.63, 3.86)	
BMI z-score at inclusion[ii]				0.50
< 50th percentile	31	118	1.04 (0.40, 2.68)	
> 50th percentile	40	102	1.67 (0.69, 4.03)	
Parental educational level[iii]				0.81
Shorter (ISCED level 1-4)	41	136	1.57 (0.67, 3.66)	
Longer (ISCED level 5-8)	24	83	1.96 (0.58, 6.62)	
Family psychiatric diagnosis[iv]				0.49
Yes	18	92	1.69 (0.48, 5.97)	
No	53	128	1.19 (0.56, 2.52)	
Immigration status[iv]				0.42
Danish Origin	45	123	1.07 (0.49, 2.32)	
Non-Danish	26	64	3.50 (0.75, 16.29)	
Change in BMI z-score[v]				0.79
Reduction	19	46	1.52 (0.38, 6.08)	
Increase	20	29	2.23 (0.42, 11.86)	
Full population	71	220	1.39 (0.74, 2.60)	
**Q2 – Feelings of loneliness at school**				
Sex[i]				0.58
Girls	32	107	0.70 (0.29, 1.72)	
Boys	38	104	0.52 (0.24, 1.16)	
BMI z-score at inclusion[ii]				0.68
< 50th percentile	31	111	0.50 (0.20, 1.25)	
> 50th percentile	39	100	0.67 (0.30, 1.48)	
Parental educational level[iii]				0.44
Shorter (ISCED level 1-4)	40	130	0.73 (0.34, 1.55)	
Longer (ISCED level 5-8)	24	80	0.38 (0.13, 1.11)	
Family psychiatric diagnosis[iv]				0.82
Yes	18	85	0.72 (0.23, 2.24)	
No	52	126	0.52 (0.25, 1.07)	
Immigration status[iv]				0.53
Danish Origin	44	118	0.61 (0.28, 1.33)	
Non-Danish	26	60	0.36 (0.12, 1.08)	
Change in BMI z-score[v]				0.67
Reduction	19	44	0.57 (0.17, 1.87)	
Increase	20	25	0.50 (0.10, 2.54)	
Full population	70	211	0.60 (0.33, 1.08)	
**Q3 – Experiences of bullying**				
Sex[i]				**0.01***
Girls	31	104	1.53 (0.63, 3.75)	
Boys	38	105	0.42 (0.19, 0.92)	
BMI z-score at inclusion[ii]				**0.05***
< 50th percentile	29	110	1.44 (0.57, 3.65)	
> 50th percentile	40	99	0.52 (0.24, 1.15)	
Parental educational level[iii]				0.35
Shorter (ISCED level 1-4)	41	129	0.66 (0.31, 1.43)	
Longer (ISCED level 5-8)	22	79	1.52 (0.55, 4.21)	
Family psychiatric diagnosis[iv]				0.22
Yes	17	88	1.17 (0.38, 3.62)	
No	52	121	0.57 (0.28, 1.17)	
Immigration status[iv]				0.81
Danish Origin	44	117	0.69 (0.32, 1.47)	
Non-Danish	25	59	0.90 (0.30, 2.65)	
Change in BMI z-score[v]				0.75
Reduction	18	42	1.01 (0.32, 3.17)	
Increase	20	26	0.62 (0.14, 2.70)	
Full population	69	209	0.76 (0.43, 1.35)	
**Q4 – Experiences of stomach aches**				
Sex[i]				0.18
Girls	31	110	0.99 (0.42, 2.33)	
Boys	38	107	0.46 (0.22, 0.99)	
BMI z-score at inclusion[ii]				0.22
< 50th percentile	29	115	0.98 (0.39, 2.46)	
> 50th percentile	40	102	0.47 (0.22, 1.02)	
Parental educational level[iii]				0.11
Shorter (ISCED level 1-4)	40	134	0.46 (0.21, 0.99)	
Longer (ISCED level 5-8)	23	82	1.27 (0.47, 3.39)	
Family psychiatric diagnosis[iv]				0.08
Yes	18	90	1.39 (0.49, 3.96)	
No	51	127	0.43 (0.21, 0.89)	
Immigration status[iv]				0.61
Danish Origin	43	122	0.74 (0.35, 1.54)	
Non-Danish	26	62	0.48 (0.17, 1.35)	
Change in BMI z-score[v]				0.93
Reduction	19	44	0.81 (0.27, 2.45)	
Increase	20	29	0.94 (0.26, 3.46)	
Full population	69	217	0.66 (0.37, 1.16)	

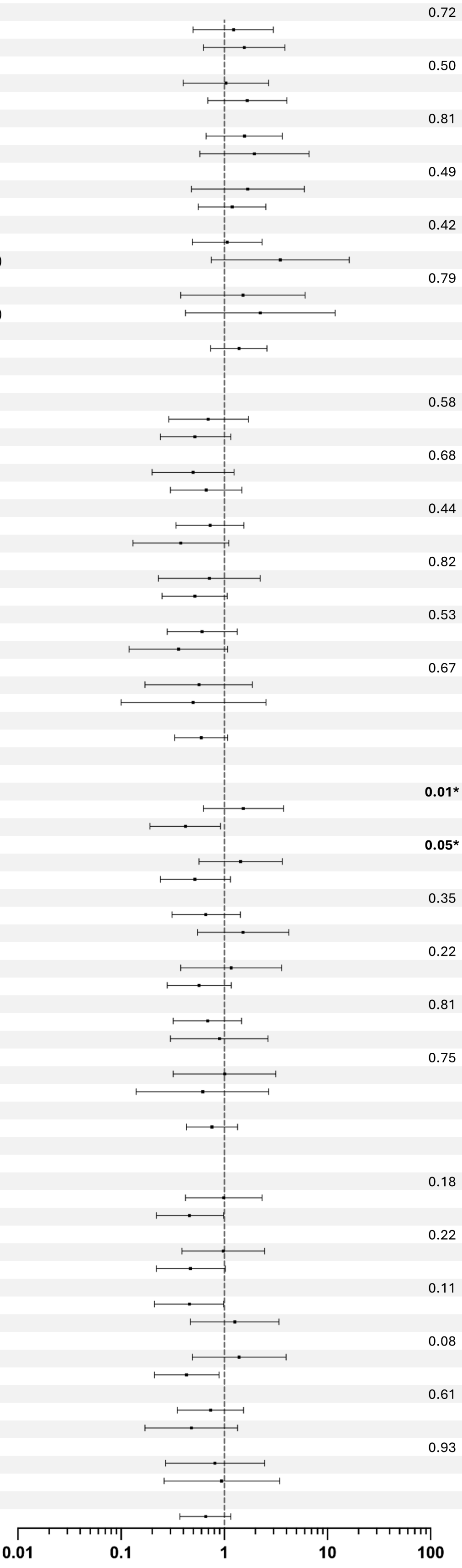

Logistic regression models comparing psychosocial well-being at follow-up between the decided-not-to-participate group and the not-invited group for the items Q1-Q4 adjusted for: [i] Well-being at inclusion, BMI z-score at inclusion, parental educational level, and immigration status. [ii] Well-being at inclusion, sex, parental educational level, and immigration status. [iii] Well-being at inclusion, BMI z-score at inclusion, sex, and immigration status. [iv] Well-being at inclusion, BMI z-score at inclusion, sex, parental educational level. [v] Well-being at inclusion, BMI z-score at inclusion, and sex. Odds ratios (ORs) greater than 1.0 indicated more favorable outcomes for the children in the decided-not-to-participate group compared with the not-invited group. *Statistically significant P-value indicating effect modification; OR, Odds ratio; CI, Confidence interval; BMI, Body mass index.

Fig. S3: Effect modification analyses comparing the psychosocial well-being between the decided-not-to-participate group and the intervention group.

	Decided-not-to-participate group (N)	Intervention group (N)	OR (95% CI)	P-value
**Q1 – Enjoyment at school**				
Sex[i]				0.93
Girls	33	54	1.39 (0.51, 3.80)	
Boys	38	48	1.26 (0.43, 3.71)	
BMI z-score at inclusion[ii]				0.43
< 50th percentile	31	52	0.94 (0.32, 2.75)	
> 50th percentile	40	50	1.77 (0.64, 4.89)	
Parental educational level[iii]				0.85
Shorter (ISCED level 1-4)	41	58	1.83 (0.69, 4.88)	
Longer (ISCED level 5-8)	24	43	1.48 (0.40, 5.47)	
Family psychiatric diagnosis[iv]				0.98
Yes	18	31	1.20 (0.28, 5.09)	
No	53	71	1.24 (0.54, 2.85)	
Immigration status[iv]				0.42
Danish Origin	45	50	1.11 (0.44, 2.81)	
Non-Danish	26	41	1.85 (0.49, 7.00)	
Change in BMI z-score[v]				0.34
Reduction	19	37	1.14 (0.27, 4.82)	
Increase	20	19	4.32 (0.74, 25.24)	
Full population	71	102	1.30 (0.63, 2.66)	
**Q2 – Feelings of loneliness at school**				
Sex[i]				**0.01***
Girls	32	55	2.73 (1.02, 7.35)	
Boys	38	48	0.49 (0.20, 1.21)	
BMI z-score at inclusion[ii]				0.41
< 50th percentile	31	53	1.55 (0.59, 4.09)	
> 50th percentile	39	50	0.85 (0.33, 2.17)	
Parental educational level[iii]				0.52
Shorter (ISCED level 1-4)	40	60	1.33 (0.56, 3.15)	
Longer (ISCED level 5-8)	24	42	0.83 (0.28, 2.52)	
Family psychiatric diagnosis[iv]				0.95
Yes	18	31	0.66 (0.14, 2.98)	
No	52	72	1.16 (0.54, 2.53)	
Immigration status[iv]				0.67
Danish Origin	44	50	1.17 (0.50, 2.73)	
Non-Danish	26	42	0.74 (0.24, 2.25)	
Change in BMI z-score[v]				0.43
Reduction	19	38	0.77 (0.24, 2.51)	
Increase	20	19	1.45 (0.33, 6.47)	
Full population	70	103	1.08 (0.56, 2.07)	
**Q3 – Experiences of bullying**				
Sex[i]				**<0.01***
Girls	31	53	6.99 (1.91, 25.52)	
Boys	38	46	0.61 (0.24, 1.53)	
BMI z-score at inclusion[ii]				0.23
< 50th percentile	29	51	2.43 (0.86, 6.87)	
> 50th percentile	40	48	1.02 (0.38, 2.71)	
Parental educational level[iii]				0.65
Shorter (ISCED level 1-4)	41	56	1.96 (0.79, 4.89)	
Longer (ISCED level 5-8)	22	42	1.55 (0.51, 4.70)	
Family psychiatric diagnosis[iv]				0.45
Yes	17	32	2.29 (0.58, 8.99)	
No	52	67	1.46 (0.62, 3.45)	
Immigration status[iv]				**<0.01***
Danish Origin	44	49	0.71 (0.29, 1.76)	
Non-Danish	25	39	9.08 (1.84, 44.77)	
Change in BMI z-score[v]				0.91
Reduction	18	36	1.98 (0.55, 7.19)	
Increase	20	16	1.03 (0.11, 9.54)	
Full population	69	99	1.48 (0.75, 2.93)	
**Q4 – Experiences of stomach aches**				
Sex[i]				0.35
Girls	31	58	1.06 (0.43, 2.64)	
Boys	38	49	0.61 (0.25, 1.49)	
BMI z-score at inclusion[ii]				0.14
< 50th percentile	29	56	1.40 (0.53, 3.72)	
> 50th percentile	40	51	0.57 (0.23, 1.41)	
Parental educational level[iii]				0.17
Shorter (ISCED level 1-4)	40	62	0.59 (0.26, 1.35)	
Longer (ISCED level 5-8)	23	44	1.38 (0.48, 3.96)	
Family psychiatric diagnosis[iv]				0.57
Yes	18	34	0.93 (0.28, 3.12)	
No	51	73	0.69 (0.32, 1.49)	
Immigration status[iv]				0.08
Danish Origin	43	53	1.35 (0.57, 3.20)	
Non-Danish	26	43	0.37 (0.12, 1.09)	
Change in BMI z-score[v]				0.97
Reduction	19	39	0.73 (0.23, 2.32)	
Increase	20	19	0.92 (0.20, 4.15)	
Full population	69	107	0.82 (0.44, 1.54)	

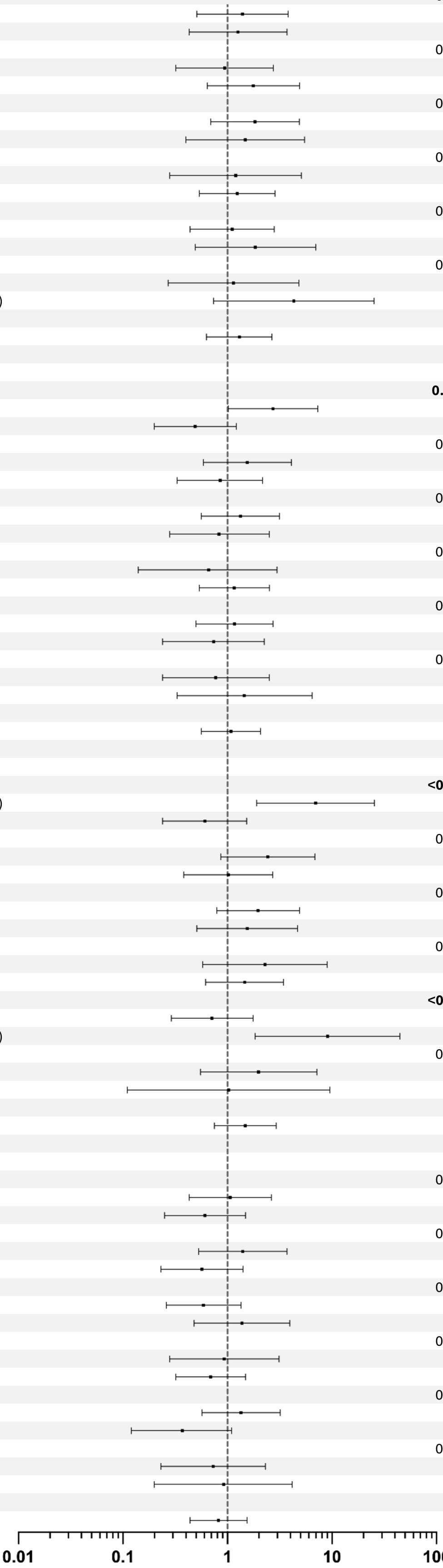

Logistic regression models comparing psychosocial well-being at follow-up between the decided-not-to-participate group and the intervention group for the items Q1-Q4 adjusted for: [i] Well-being at inclusion, BMI z-score at inclusion, parental educational level, and immigration status. [ii] Well-being at inclusion, sex, parental educational level, and immigration status. [iii] Well-being at inclusion, BMI z-score at inclusion, sex, and immigration status. [iv] Well-being at inclusion, BMI z-score at inclusion, sex, parental educational level. [v] Well-being at inclusion, BMI z-score at inclusion, and sex. Odds ratios (ORs) greater than 1.0 indicated more favorable outcomes for the children in the decided-not-to-participate group compared with the intervention group. *Statistically significant P-value indicating effect modification; OR, Odds ratio; CI, Confidence interval; BMI, Body mass index.
